# Toll-Like Receptor Homolog CD180 Expression Is Diminished on Natural Autoantibody-Producing B Cells of Patients with Autoimmune CNS Disorders

**DOI:** 10.1155/2021/9953317

**Published:** 2021-05-25

**Authors:** Zsófia Hayden, Szabina Erdő-Bonyár, Beáta Bóné, Noémi Balázs, Kornélia Bodó, Zsolt Illes, Timea Berki, Diána Simon

**Affiliations:** ^1^Department of Immunology and Biotechnology, Clinical Center, University of Pécs Medical School, H-7624 Pécs, Hungary; ^2^Department of Neurology, Clinical Center, University of Pécs Medical School, H-7623 Pécs, Hungary; ^3^Department of Neurology, Odense University Hospital, Denmark; ^4^Brain Research Inter-Disciplinary Guided Excellence, Department of Clinical Research, Faculty of Health Sciences, University of Southern Denmark, Denmark

## Abstract

**Purpose:**

Decreased expression of TLR homolog CD180 in peripheral blood B cells and its potential role in antibody production have been described in autoimmune diseases. Effectiveness of anti-CD20 therapy in neuromyelitis optica spectrum disorder (NMOSD) and multiple sclerosis (MS) strengthens the role of B cells in the pathogenesis. Therefore, we aimed to investigate the CD180 expression of peripheral blood B cell subsets in NMOSD and MS patients and analyze the levels of natural anti-citrate synthase (CS) IgG autoantibodies and IgG antibodies induced by bacterial infections reported to play a role in the pathogenesis of NMOSD or MS.

**Methods:**

We analyzed the distribution and CD180 expression of peripheral blood B cell subsets, defined by CD19/CD27/IgD staining, and measured anti-CS IgM/G natural autoantibody and antibacterial IgG serum levels in NMOSD, RRMS, and healthy controls (HC).

**Results:**

We found decreased naïve and increased memory B cells in NMOSD compared to MS. Among the investigated four B cell subsets, CD180 expression was exclusively decreased in CD19^+^CD27^+^IgD^+^ nonswitched (NS) memory B cells in both NMOSD and MS compared to HC. Furthermore, the anti-CS IgM natural autoantibody serum level was lower in both NMOSD and MS. In addition, we found a tendency of higher anti-CS IgG natural autoantibody levels only in anti-Chlamydia IgG antibody-positive NMOSD and MS patients.

**Conclusions:**

Our results suggest that reduced CD180 expression of NS B cells could contribute to the deficient natural IgM autoantibody production in NMOSD and MS, whereas natural IgG autoantibody levels show an association with antibacterial antibodies.

## 1. Introduction

Multiple sclerosis (MS) is a chronic, progressive, neuroinflammatory disease characterized by immune-mediated inflammation, demyelination, and axonal damage in the central nervous system (CNS) [1–3]. Neuromyelitis optica spectrum disorder (NMOSD) is an inflammatory autoimmune disease of the CNS, primarily affecting the optic nerves and the spinal cord, leading to blindness and paralysis [4–7]. NMOSD was only recognized as a distinct disease entity and separated from MS over the past 10 years with the discovery of a unique biomarker, autoantibodies against the aquaporin-4 (AQP4) molecule [8]. However, 10-25% of patients with a clinical diagnosis of NMOSD remain AQP4 antibody-negative [9]. In addition to antibody production, B cells are important in antigen presentation and proinflammatory cytokine secretion [10]. The clinical success of anti-CD20 antibodies in the treatment of MS and NMOSD [11, 12] underlines the important role of B cells in disease initiation and progression. Studies focusing on B cell subpopulations in MS and NMOSD are limited, and the precise role and changes in naïve and memory B cell distribution are still unclear in the development of MS and NMOSD.

CD180, or RP105 (radioprotective 105 kDa), is a Toll-like receptor (TLR) homolog molecule expressed by B cells, monocytes, and dendritic cells, and it mediates polyclonal B cell activation, proliferation, and immunoglobulin production [13, 14]. The altered expression and functions of CD180 in B cells have been described in autoimmune diseases [13]. CD180-negative B cells were increased in patients with Sjögren's syndrome [15] and in systemic lupus erythematosus (SLE) patients [16]. Moreover, disease severity in SLE correlated with the amount of CD180-negative B cells in the peripheral blood [17, 18]. In our previous study [19], we found significantly lower CD180 expression in peripheral blood B cells of early diffuse cutaneous systemic sclerosis (dcSSc) patients. We also found that nonswitched (NS) memory B cells showed the strongest activation after CD180 ligation, and stimulation via CD180 resulted in enhanced natural autoantibody production by tonsillar B cells.

In our previous studies [20, 21], we have detected natural antibodies recognizing anti-citrate synthase (CS) in healthy controls (HC) and patients with systemic autoimmune diseases. Monitoring of anti-CS IgM autoantibodies in healthy adults over a five-year period showed that the titer of anti-CS IgM antibodies is constant and characteristic for the given individual [20]. We measured significantly higher levels of anti-CS IgM autoantibodies in anti-dsDNA IgM-positive SLE serum samples; besides, anti-CS IgM and anti-dsDNA IgM levels also showed correlation, supporting that these IgM autoantibodies are part of the natural immune repertoire in SLE patients [22]. According to our previous studies, the titer of anti-CS IgG antibodies is fluctuating over time [20], and it shows an association with infection-induced antibodies [23].

In this study, we sought to investigate the distribution and CD180 expression of peripheral blood B cell subsets, defined by CD27 and IgD staining in NMOSD and MS patients, and correlate the levels of natural anti-CS IgG with IgG antibody titers induced by bacterial infections described to play a role in the pathogenesis of NMOSD or MS.

## 2. Materials and Methods

### 2.1. Patients

Fifteen patients with relapsing-remitting multiple sclerosis (RRMS), twelve patients with neuromyelitis optica spectrum disorder (NMOSD), and six age- and sex-matched healthy controls (HC) were enrolled in the study. All RRMS patients met the revised McDonald criteria, and all NMOSD patients were diagnosed based on the 2015 new diagnostic criteria for NMOSD. All 12 NMOSD patients included in our study were treated with immunosuppressive drugs, including steroid, azathioprine, or tocilizumab, and were considered to be treatment responders. From the total 15 RRMS patients included in our study, 14 patients were considered to be treatment responders. One RRMS patient was considered a treatment nonresponder, and following peripheral blood sample taking, the patient's therapy was changed from fingolimod to natalizumab. Clinical samples were obtained with patients' informed consent. The study was approved by the Regional Research Ethics Committee of the Medical Center, University of Pécs (RIKEB 7954/2019). Detailed patient data are summarized in Table 1.

### 2.2. Flow Cytometric Analysis

To analyze the distribution of peripheral blood naïve and memory B cell subsets (NMOSD *n* = 12, MS *n* = 15, and HC *n* = 6) and to evaluate their CD180 expression (NMOSD *n* = 9, MS *n* = 7, and HC *n* = 5) by flow cytometry, four-color analysis was conducted using the combination of anti-human CD19-FITC (4G7, BD Biosciences Pharmingen, San Diego, CA, USA), anti-human CD27-APC (M-T271, BD Biosciences Pharmingen, San Diego, CA, USA), anti-human IgD-PerCP (IA6-2, BioLegend, San Diego, CA, USA), and anti-CD180-PE (G28-8, Becton Dickinson, Franklin Lakes, NJ, USA) antibodies, following the manufacturer's instructions. Briefly, peripheral blood samples were incubated with antibodies for 20 min. After hemolysis, cells were washed in phosphate-buffered saline (PBS) and fixed with FACSFix (0.5% PFA in PBS). Fluorescence of labeled cells was recorded using BD FACSCalibur (BD Biosciences Pharmingen, San Diego, CA, USA) and analyzed with FCS Express 6 software (De Novo Software, Pasadena, CA, USA).

### 2.3. Naïve and Memory B Cell Separation

Peripheral blood mononuclear cells (PBMCs) were isolated using the Ficoll-Paque Plus density gradient centrifugation of peripheral blood samples (NMOSD *n* = 5, MS *n* = 5, and HC *n* = 5). PBMCs were washed twice in PBS and incubated with anti-human CD19-FITC (4G7, BD Biosciences Pharmingen, San Diego, CA, USA) and anti-human CD27-APC (M-T271, BD Biosciences Pharmingen, San Diego, CA, USA) antibodies for 30 min at 4°C, following the manufacturer's instructions. After the incubation period, samples were washed twice in PBS and taken up in an in-house buffer solution (containing PBS 1x, 0.5% BSA, and 0.75% EDTA) and filtered through a cell strainer cap into Falcon polystyrene tubes under sterile conditions. Separation of naïve (CD19^+^CD27^−^) and memory (CD19^+^CD27^+^) B cells was performed using the S3e Cell Sorter (Life Science Research/Bio-Rad, Hercules, CA, USA). The purity of naïve and memory B cell populations was checked using the BD FACSCalibur flow cytometer.

### 2.4. RNA Isolation, cDNA Synthesis, and qPCR

Total RNA was extracted from naïve (CD19^+^CD27^−^) and memory (CD19^+^CD27^+^) B cells immediately after their separation using the NucleoSpin RNA XS kit (Macherey-Nagel Inc., Bethlehem, PA, USA). Next, cDNA was generated with the High-Capacity cDNA Reverse Transcription Kit (Thermo Fisher Scientific, Waltham, MA, USA), and CD180 mRNA expression of naïve and memory B cells (NMOSD *n* = 5, MS *n* = 5, and HC *n* = 5) was determined by qPCR using the SensiFAST SYBR Lo-ROX Kit (Bioline, London, UK). Amplifications were performed using the Applied Biosystems 7500 RT-PCR System (Thermo Fisher Scientific, Waltham, MA, USA), and CD180 gene expression was analyzed using 7500 Software v2.0.6 (Thermo Fisher Scientific, Waltham, MA, USA). The mRNA expression of CD180 was normalized to GAPDH (a “housekeeping” gene) as a reference, and fold changes (RQ) were calculated based on the 2-ddCT method.

### 2.5. Measurement of Anti-citrate Synthase Antibodies

Anti-citrate synthase (CS) IgG/M levels were measured with in-house ELISA as described earlier [22]. Briefly, Nunc MaxiSorp™ ELISA plates were coated with citrate synthase from porcine hearts (Sigma-Merck C3260) at a concentration of 2.25 *μ*g/mL in a coating buffer (Bio-Rad BUF030) (50 *μ*L/well, 4-6°C, and overnight). After blocking with 0.5 m/m% PVA (~72,000 Mw, 300 *μ*L/well, room temperature, and ≥2 hours), serum samples (NMOSD *n* = 10, MS *n* = 13, and HC *n* = 5) were incubated in 100-fold dilution in a washing buffer (WB) (100 mM PBS, pH 7.4 + 1 mL/L Tween 20) for 35 min at room temperature (RT) (standards, blanks, and high and low controls were processed as patient sera). After 3 washing steps, the anti-human IgM or IgG secondary antibody (Dako) was incubated for 30 min, followed by the 3,3′,5,5′-tetramethylbenzidine (TMB) substrate for 15 min and H_2_SO_4_ stop solution (50 *μ*L/well), and reading was performed at *λ* = 450/620 using a Siemens BEP 2000 Advance® platform (Siemens AG, Frankfurt, Germany). Five-point dilution series of our in-house anti-CS standard was used for result quantitation, with subsequent 4-point sigmoid curve fitting.

### 2.6. Detection of Antibacterial Antibodies

Commercial ELISA kits were used to detect infection-induced antibodies in sera. Anti-Chlamydia pneumoniae IgM/G/A, anti-Chlamydia trachomatis IgM/G/A (NovaLisa, NovaTec GmbH, Dietzenbach, Germany), anti-Mycoplasma pneumoniae IgM/G/A (VIROTECH Diagnostics GmbH, Rüsselsheim, Germany), anti-Helicobacter pylori IgG/A, and anti-Borrelia burgdorferi IgM/G (Mikrogen GmbH, Neureid, Germany) autoantibodies were measured, according to the manufacturer's instructions. Briefly, serum samples at 1 : 100 dilution were incubated for 1 hour at RT. Subsequently, plates were incubated with horseradish peroxidase- (HRP-) conjugated anti-human IgA/IgG/IgM antibodies for 30 min at RT. Color reaction was developed with TMB. Finally, stop solution was applied, and optical density was detected at 450 nm using a Siemens BEP 2000 Advance® platform (Siemens AG, Frankfurt, Germany).

### 2.7. Statistical Analysis

Statistical evaluation was performed with the SPSS IBM version 26 statistics package (IBM, Armonk, NY, USA). Student's *t*-tests, ANOVA, Mann-Whitney *U* tests, and Kruskal-Wallis tests were used as appropriate, and *p* values < 0.05 were considered statistically significant.

## 3. Results

### 3.1. Increased Memory and Decreased Naïve B Cell Ratios in NMOSD Compared to MS

**Figure 1:**
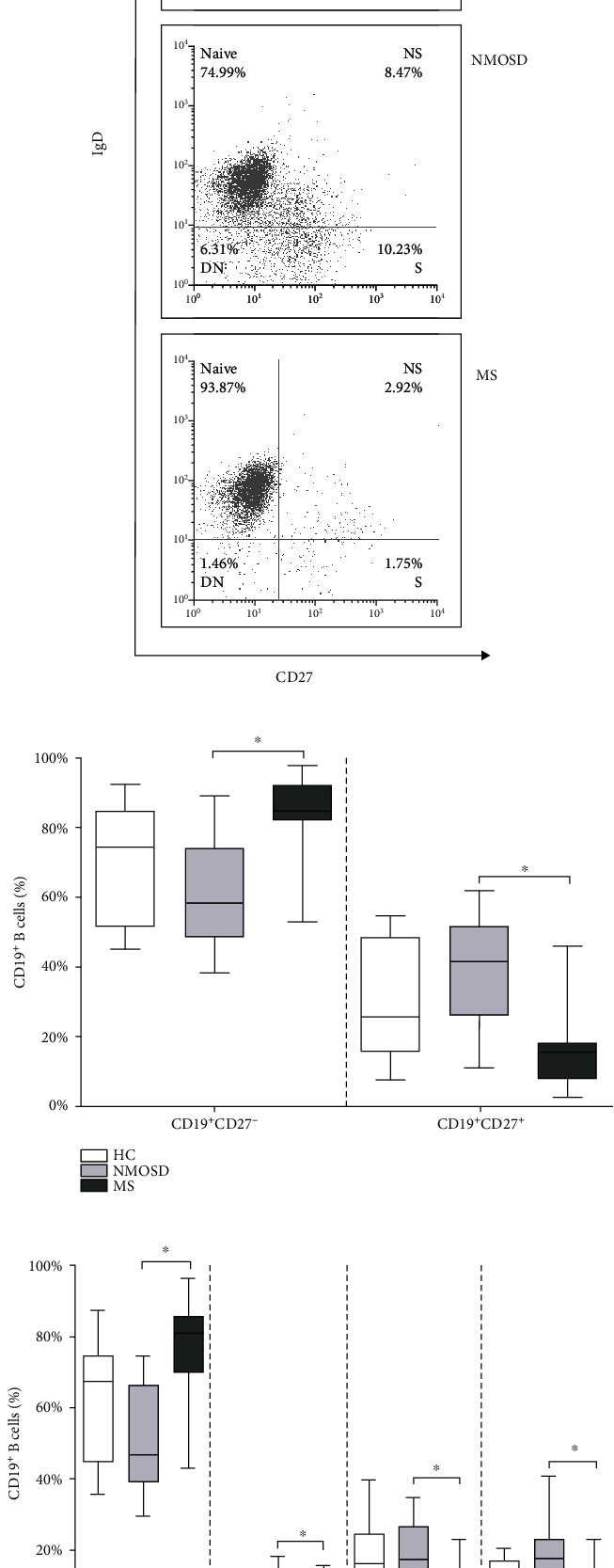
Analysis of B cell subsets in neuromyelitis optica spectrum disorder (NMOSD) and multiple sclerosis (MS) patients. (a) Representative flow cytometry plots of four subsets of peripheral blood CD19^+^ B cells defined by CD27 and IgD labeling: CD19^+^CD27^−^IgD^+^ naïve, CD19^+^CD27^+^IgD^+^ nonswitched (NS) memory, CD19^+^CD27^+^IgD^−^ switched (S) memory, and CD19^+^CD27^−^IgD^−^ double-negative (DN) B cells. (b) Flow cytometric analysis of naïve (CD19^+^CD27^−^) and memory (CD19^+^CD27^+^) B cells in peripheral blood of NMOSD, MS, and healthy controls (HC). (c) Flow cytometric analysis of the defined four B cell subpopulations in peripheral blood of NMOSD, MS, and healthy controls (HC). Boxes show interquartile ranges (IQR); whiskers indicate the lowest and highest values; horizontal lines represent medians; *n* = 6 HC, *n* = 12 NMOSD, and *n* = 15 MS; ^∗^*p* < 0.05.

First, we analyzed percentages of total CD19^+^ B cells in NMOSD and MS, which showed no significant differences compared to HC (NMOSD *n* = 12, median: 6.3, and range: 1-20.7; MS *n* = 15, median: 7.3, and range: 0.5-32.7; and HC *n* = 6, median: 7.3, and range: 3.8-12.7). Next, we compared the distribution of naïve and memory B cell subsets in NMOSD and MS. We used CD19 as a lineage marker of B cells [24] and CD27, which is considered a universal memory B cell marker. Naïve B cells were characterized by the lack of CD27 expression [25]. The ratio of naïve (CD19^+^CD27^−^) and memory (CD19^+^CD27^+^) B cells in NMOSD and MS showed no significant differences compared to HC. However, in NMOSD, the frequency of naïve (CD19^+^CD27^−^) B cells was significantly lower, and the percentage of memory (CD19^+^CD27^+^) B cells was significantly higher compared to MS (Figure 1(b)).

To analyze the distribution of memory B cell subsets, four B cell subpopulations were defined by CD27 and IgD labeling: CD19^+^CD27^+^IgD^−^ switched (S) memory B cells, CD19^+^CD27^+^IgD^+^ nonswitched (NS) memory B cells, CD19^+^CD27^−^IgD^+^ naïve B cells, and CD19^+^CD27^−^IgD^−^ double-negative (DN) B cells (Figure 1(a)). We found a significantly lower percentage of naïve and higher frequency of NS, S, and DN B cells in NMOSD compared to MS (Figure 1(c)).

To investigate the potential effect of therapy on the distribution of B cell subsets, we compared the proportion of B cell subpopulations between immunomodulatory treated (*n* = 10) and untreated (*n* = 4) MS patients and found no differences (data not shown). Similar measurements were not applicable in NMOSD as all patients received immunosuppressive drugs.

### 3.2. CD180 Expression of Nonswitched Memory B Cells Is Lower in NMOSD and MS Patients Than in HC

**Figure 2:**
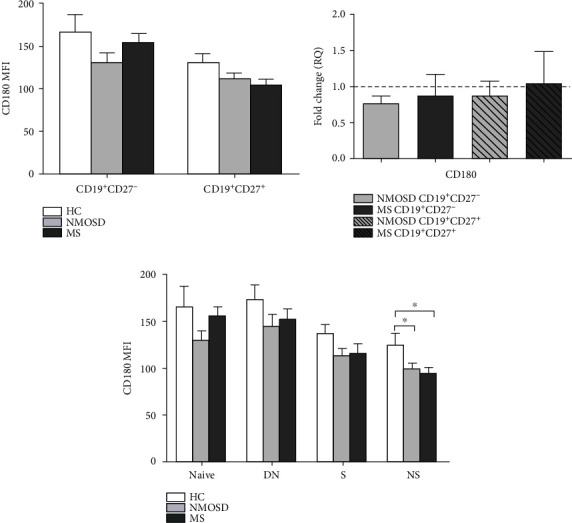
Analysis of CD180 expression in B cell subsets of neuromyelitis optica spectrum disorder (NMOSD) and multiple sclerosis (MS) patients. (a) Flow cytometric analysis of CD180 expression in naïve (CD19^+^CD27^−^) and memory (CD19^+^CD27^+^) B cells in peripheral blood of NMOSD (*n* = 9), MS (*n* = 7), and healthy controls (HC) (*n* = 5). (b) CD180 mRNA expression in B cells of NMOSD (*n* = 5) and MS (*n* = 5) patients compared to HC (*n* = 5). Gene expression was normalized to HC, and the horizontal line (value 1) represents the expression of control samples. Changes in gene expression are shown as relative quantification (RQ) values. (c) Flow cytometric analysis of CD180 expression in CD19^+^CD27^−^IgD^+^ naïve, CD19^+^CD27^−^IgD^−^ double-negative (DN), CD19^+^CD27^+^IgD^−^ switched (S) memory, and CD19^+^CD27^+^IgD^+^ nonswitched (NS) memory B cells in peripheral blood of NMOSD (*n* = 9), MS (*n* = 7), and healthy controls (HC) (*n* = 5). Data are shown as mean ± standard error of the mean (SEM); ^∗^*p* < 0.05.

Since altered expression of CD180 in autoimmune diseases and its potential pathological role in B cell activation and autoantibody production were already described [15–18], we measured CD180 expression at protein (mean fluorescence intensity (MFI)) and mRNA (RQ) levels in naïve (CD19^+^CD27^−^) and memory (CD19^+^CD27^+^) B cell subsets. We found no significant differences in any investigated B cell subsets among NMOSD, MS, and HC (Figures 2(a) and 2(b)).

Next, we analyzed the MFI of CD180 expression in CD19^+^CD27^−^IgD^+^ naïve, CD19^+^CD27^+^IgD^+^ nonswitched (NS) memory, CD19^+^CD27^+^IgD^−^ switched (S) memory, and CD19^+^CD27^−^IgD^−^ double-negative (DN) B cells and found a significantly decreased level of CD180 expression in NS B cells of both the NMOSD and MS patients compared to HC (Figure 2(c)).

### 3.3. IgM Natural Autoantibody Level Is Diminished in Both the NMOSD and MS Patients Compared to HC

**Figure 3:**
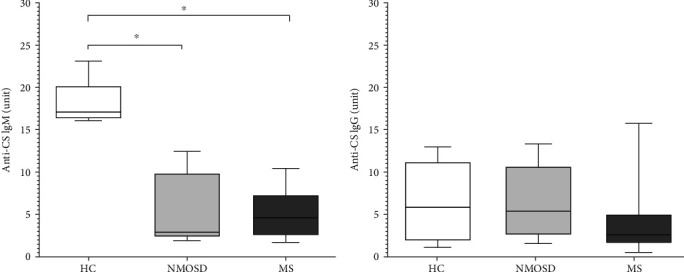
Natural autoantibody serum levels in neuromyelitis optica spectrum disorder (NMOSD) and multiple sclerosis (MS) patients. Anti-citrate synthase (CS) IgM (a) and IgG (b) levels in healthy controls (HC), NMOSD, and MS as measured by ELISA. Boxes show interquartile ranges (IQR); whiskers indicate the lowest and highest values; horizontal lines represent medians; *n* = 5 HC, *n* = 10 NMOSD, and *n* = 13 MS; ^∗^*p* < 0.05.

In our previous study [19], we found that B cell stimulation via CD180 resulted in strong activation of NS B cells, along with a significant decrease in their CD180 expression and induction of natural autoantibody production. We also described alterations in natural autoantibody (anti-citrate synthase (CS)) levels in patients with different systemic autoimmune diseases [22]. Consequently, we measured anti-CS IgM/G natural autoantibody levels in sera of patients with NMOSD or MS and HC. The anti-CS IgM level was significantly decreased in NMOSD and MS samples compared to HC (Figure 3(a)), but no differences were found in anti-CS IgG levels (Figure 3(b)).

### 3.4. Anti-CS IgG Natural Autoantibody Level Is Elevated in Anti-Chlamydia pneumoniae IgG-Positive Patients

Since we previously found associations between antibacterial antibodies and IgG natural autoantibodies in various autoimmune diseases [22, 23] and several infections, including Chlamydia pneumoniae, Chlamydia trachomatis, Mycoplasma pneumoniae, Helicobacter pylori, and Borrelia burgdorferi [26], are reported to have a potential role in the development of NMOSD or MS, we measured IgM, IgG, and IgA antibodies directed against these pathogens. We found that anti-Chlamydia pneumoniae IgG was detected in 54.5% (6/11) of NMOSD patients and in 14.3% (2/14) of MS patients. Anti-Mycoplasma pneumoniae IgG was detected in 9.1% (1/11) of NMOSD patients, and anti-Mycoplasma pneumoniae IgG/A was found in 28.5% (4/14) of MS patients. Anti-Helicobacter pylori IgG/A was detected in 36.4% (4/11) of NMOSD patients and in 14.3% (2/14) of MS patients. Anti-Borrelia burgdorferi IgM was detected in 7.1% (1/14) of MS patients. Neither of the NMOSD or MS patients was positive for anti-Chlamydia trachomatis IgM/G/A.

**Figure 4:**
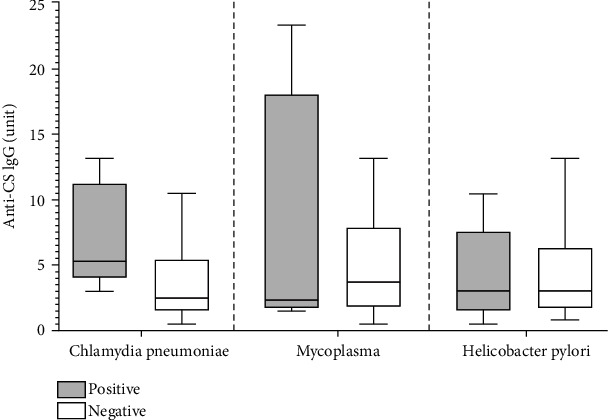
Natural IgG autoantibody levels in neuromyelitis optica spectrum disorder (NMOSD) and multiple sclerosis (MS) patients positive or negative for the investigated antibacterial IgG antibodies. Anti-citrate synthase (CS) IgG antibody levels in anti-Chlamydia pneumoniae, anti-Mycoplasma pneumoniae, and anti-Helicobacter pylori IgG-positive and IgG-negative NMOSD and MS patients, as measured by ELISA. Boxes show interquartile ranges (IQR); whiskers indicate the lowest and highest values; horizontal lines represent medians; *n*_NMOSD+MS_ = 25.

We also analyzed the relationship between anti-citrate synthase (CS) IgM or IgG natural autoantibody levels and antibacterial antibody positivity in NMOSD and MS patients. We found a higher tendency of anti-CS IgG levels in the anti-Chlamydia pneumoniae IgG-positive patients compared to the anti-Chlamydia pneumoniae IgG-negative patients, but we did not find differences between the anti-Mycoplasma pneumoniae IgG-positive and anti-Mycoplasma pneumoniae IgG-negative patients and the anti-Helicobacter pylori IgG-positive and anti-Helicobacter pylori IgG-negative patients (Figure 4).

## 4. Discussion

Several studies focus on the distribution of B cell subpopulations in MS and NMOSD, whereas the functional characterization of B cell subsets in these disorders is limited. In this study, we found no significant differences in the percentage of total CD19^+^ B cells and distribution of B cell subsets in NMOSD or MS compared to HC. This is in agreement with previous findings [27–29] reporting no significant differences in the distribution of naïve and memory B cell subsets in MS compared to HC. However, the decreased percentage of total CD19^+^ B cells in RRMS patients [30] and increased proportion of memory B cells in untreated MS patients compared to HC have also been reported [1, 31]. Several studies [32] reported altered distribution of B cell subsets in MS patients treated with different disease-modifying therapies (DMT). Increased proportion of memory B cells was described in MS patients treated with natalizumab [33] or atacicept [34], whereas reduced proportion of memory B cells was reported in MS patients treated with dimethyl fumarate [35], interferon *β* [36], glatiramer acetate [37], fingolimod [38], and alemtuzumab. Similar to our study, Habib et al. [27] did not observe any significant differences related to the type of disease-modifying therapies (DMT) that MS patients received. The effect of immunosuppressive therapies on alterations of B cell subsets in NMOSD and MS patients has also been investigated. Janssen et al. [5] reported significantly elevated levels of naïve B cell ratios in NMOSD compared to HC. Kowarik et al. [39] reported significantly elevated DN B cells and significantly lower memory B cells in NMOSD (*n* = 7) compared to MS (*n* = 15), and there were no significant differences in the proportion of NMOSD naïve B cells compared to MS and HC. We also found the significantly elevated ratio of DN B cells in NMOSD compared to MS, and there were no differences in the proportion of NMOSD naïve B cells compared to HC; however, we found significantly increased frequencies of both the S and NS memory B cells in NMOSD compared to MS.

The TLR homolog CD180 molecule activates the majority of B cells, resulting in phenotypic and functional alterations [40–42]. Distinct expression and functions of CD180 on B cells have been associated with infection, chronic inflammation, and autoimmune diseases [13, 19]. Increased proportion of CD180-negative B cells was described in SLE [16] and Sjögren's syndrome, and we previously reported [19] significantly decreased expression of CD180 in B cells of dcSSc patients. In this study, we found that the expression of CD180 was exclusively decreased in NS B cells in NMOSD and MS compared to HC. It was already described in SLE that the CD180-negative B cells are highly activated cells [16], and we previously reported that anti-CD180 antibody ligation resulted in decreased CD180 expression; thus, the diminished CD180 expression of NS memory B cells in NMOSD and MS might be a result of B cell activation via CD180. NS B cells resemble B1 B cells [19, 43] and have innate-like features, suggesting their potential role in natural autoantibody production. The majority of natural autoantibodies are of IgM isotype, polyreactive, and low-titer antibodies, and their presence in infants and their unaltered serum level during ≥5 years in adults indicate that these antibodies belong to the natural autoantibody repertoire established early in postnatal life [20, 21]. They participate in the removal of apoptotic cells, leading to a decrease of inflammation, also maintain tissue homeostasis and immunological balance, and can prevent the development of autoimmunity [44, 45]. We previously showed that NS B cells are highly activated by CD180 ligation resulting in the enhancement of natural IgM autoantibody production [19]. According to our results, diminished CD180 expression of NS B cells could contribute to lower anti-CS IgM levels found in NMOSD and MS compared to HC. Our observation supports the *in vivo* therapeutic efficacy of IVIgM [46], which was confirmed in experimental models of uveitis, myasthenia gravis, and MS [47, 48].

We previously reported a correlation between anti-CS IgG levels and cardiovascular disease-associated pathogens, including Chlamydia pneumoniae in coronary artery bypass grafting patients [23] and higher anti-CS IgG levels in anti-measles IgG-positive SLE patients [22], indicating a connection between natural IgG autoantibodies and infection-induced antibodies. Since data have been published on the possible involvement of Chlamydia pneumoniae, Chlamydia trachomatis, Mycoplasma pneumoniae, Helicobacter pylori, and Borrelia burgdorferi in the development of NMOSD or MS [26], we investigated the relationship between these antibacterial antibodies and natural autoantibodies. We found a higher tendency of anti-CS IgG levels in anti-Chlamydia pneumoniae IgG-positive NMOSD and MS patients than in anti-Chlamydia pneumoniae IgG-negative patients. The titer of natural IgG autoantibodies fluctuates over time, they are abundant in human sera, and their levels are influenced by age, gender, and disease, indicating that their presence may be due to adaptive-like immune responses [49, 50].

In conclusion, our results support the role of B cell subsets in the fine-tuning of immune homeostasis. We highlight the importance of natural autoantibodies, the first-line components of the adaptive immune response in the balance of self-tolerance and antimicrobial immunity and in the development of autoimmune diseases of the CNS.

## Figures and Tables

**Table 1:** Clinical characteristics of neuromyelitis optica spectrum disorder (NMOSD) patients, relapsing-remitting multiple sclerosis (RRMS) patients, and healthy controls (HC) involved in the study.

	HC (*n* = 6)	NMOSD (*n* = 12)	RRMS (*n* = 15)
Gender (female), *n* (%)	4 (66.7%)	7 (58.3%)	13 (86.7%)
Anti-AQP4 antibody positivity, *n* (%)	—	8 (66.7%)	—
Median age, *y* (range)	47.5 (25-52)	50.5 (30-71)	42 (22-65)
Median age at onset, *y* (range)	—	44 (16-69)	28 (12-42)
Median disease duration, *y* (range)	—	8 (0.5-22)	15 (1-34)
No. of relapse, mean ± SD	—	2.5 ± 1.1	3.2 ± 1.8
EDSS, median (range)	—	2 (0-6.5)	1.5 (0-8)
DMT drug^∗^	—	—	10 (66.7%)
Immunosuppressive drug	—	12 (100%)	1 (6.7%)^♦^

^∗^DMT drugs in MS included dimethyl fumarate (4), interferon *β*1a (2), fingolimod (2), glatiramer acetate (1), and alemtuzumab (1). HC = healthy controls; NMOSD = neuromyelitis optica spectrum disorder; MS = multiple sclerosis; AQP4 = aquaporin-4; EDSS = Expanded Disability Status Scale; DMT = disease-modifying therapies. ^♦^One RRMS patient received immunosuppressive therapy due to the first attack of the disease; first, high-dose parenteral steroid therapy was applied, followed by oral steroid treatment, which was ceased due to visual improvement of the patient.

## Data Availability

The data that support the findings of this study are available from the authors (Hayden Z and Simon D) upon reasonable request.
